# Mitochondrial Localization of ABC Transporter ABCG2 and Its Function in 5-Aminolevulinic Acid-Mediated Protoporphyrin IX Accumulation

**DOI:** 10.1371/journal.pone.0050082

**Published:** 2012-11-26

**Authors:** Hirotsugu Kobuchi, Koko Moriya, Tetsuya Ogino, Hirofumi Fujita, Keiji Inoue, Taro Shuin, Tatsuji Yasuda, Kozo Utsumi, Toshihiko Utsumi

**Affiliations:** 1 Department of Cell Chemistry, Okayama University Graduate School of Medicine, Dentistry and Pharmaceutical Sciences, Okayama, Japan; 2 Applied Molecular Bioscience, Graduate School of Medicine, Yamaguchi University, Yamaguchi, Japan; 3 Department of Nursing Science, Faculty of Health and Welfare Science, Okayama Prefectural University, Soja, Japan; 4 Department of Cytology and Histology, Okayama University Graduate School of Medicine, Dentistry and Pharmaceutical Sciences, Okayama, Japan; 5 Department of Urology, Kochi Medical School, Nankoku, Kochi, Japan; University of Pecs Medical School, Hungary

## Abstract

Accumulation of protoporphyrin IX (PpIX) in malignant cells is the basis of 5-aminolevulinic acid (ALA)-mediated photodynamic therapy. We studied the expression of proteins that possibly affect ALA-mediated PpIX accumulation, namely oligopeptide transporter-1 and -2, ferrochelatase and ATP-binding cassette transporter G2 (ABCG2), in several tumor cell lines. Among these proteins, only ABCG2 correlated negatively with ALA-mediated PpIX accumulation. Both a subcellular fractionation study and confocal laser microscopic analysis revealed that ABCG2 was distributed not only in the plasma membrane but also intracellular organelles, including mitochondria. In addition, mitochondrial ABCG2 regulated the content of ALA-mediated PpIX in mitochondria, and Ko143, a specific inhibitor of ABCG2, enhanced mitochondrial PpIX accumulation. To clarify the possible roles of mitochondrial ABCG2, we characterized stably transfected-HEK (ST-HEK) cells overexpressing ABCG2. In these ST-HEK cells, functionally active ABCG2 was detected in mitochondria, and treatment with Ko143 increased ALA-mediated mitochondrial PpIX accumulation. Moreover, the mitochondria isolated from ST-HEK cells exported doxorubicin probably through ABCG2, because the export of doxorubicin was inhibited by Ko143. The susceptibility of ABCG2 distributed in mitochondria to proteinase K, endoglycosidase H and peptide-*N*-glycosidase F suggested that ABCG2 in mitochondrial fraction is modified by *N*-glycans and trafficked through the endoplasmic reticulum and Golgi apparatus and finally localizes within the mitochondria. Thus, it was found that ABCG2 distributed in mitochondria is a functional transporter and that the mitochondrial ABCG2 regulates ALA-mediated PpIX level through PpIX export from mitochondria to the cytosol.

## Introduction

Photodynamic diagnosis (PDD) and photodynamic therapy (PDT) is a promising local treatment modality based on the selective accumulation of a photosensitizer in malignant tissue [1], [2]. 5-Aminolevulinic acid (ALA) is the naturally occurring metabolic precursor of an endogenously synthesized photosensitizer, protoporphyrin IX (PpIX) [2]–[4]. Exogenously administered ALA-mediated PDD and PDT were proved to be clinically useful for the treatment of various carcinomas [5]–[7]. Despite the importance of PpIX in ALA-mediated PDD and PDT, the mechanism of accumulation of this metabolite in cancer cells remains unclear. The intracellular localization and concentrations of protoporphyrins are determined by at least three rate-limiting steps, namely ALA uptake through proton-coupled oligopeptide transporters, such as PEPT1 and PEPT2 [8]–[10], conversion of PpIX to heme (ferrochelatase, FECH) [11], [12], and intracellular traffic of PpIX through ATP-binding cassette (ABC) transporter G2 (ABCG2) [13], [14]. ABCG2, also called breast cancer resistant protein, was first discovered in doxorubicin-resistant breast cancer cells [15], and it predominantly localizes to the plasma membrane [16]. Accumulating evidence indicates that ABCG2 plays a particularly important role in regulating the cellular accumulation of porphyrin derivatives in cancer cells, and thereby affects the efficacy of PDD and PDT [17]. With respect to protoporphyrin traffic, we reported previously the serum-dependent export of PpIX by ABCG2 in T24 cells [18]. In this context, it was reported that ABC transporters in mitochondria also have important roles in the intracellular traffic of heme and various protoporphyrin intermediates of heme synthesis [19]. Furthermore, it has been shown recently that ABCG2 was distributed in mitochondria and functioned as a regulatory protein in multidrug resistant cells [20]. To date, four ABC transporters have been identified in mitochondria, most of which act as exporters [19]. Accumulating evidence indicates that ABCB7/ATM1, ABCB10/MDL1 and ABCB8 are localized in the inner mitochondrial membrane, whereas ABCB6 is found in the outer mitochondrial membrane. ABCB6 mediates the uptake of coproporphyrinogen III into the mitochondria, and ABCB7 and ABCB10 mediate the efflux of iron and toxic reagents into the cytosol, respectively, whereas the detailed function of ABCB8 is not well analyzed [19]. Furthermore, although the transport processes that mediate the uptake and efflux of heme and porphyrin have been studied, it still remains unknown whether ABCG2 distributed in the mitochondrial fraction is functional for the efflux of PpIX from mitochondria. In the present study, we described that functionally active ABCG2 is distributed in mitochondria and that Ko143, a specific inhibitor of ABCG2, increased the accumulation of ALA-mediated PpIX in mitochondria.

**Figure 1:**
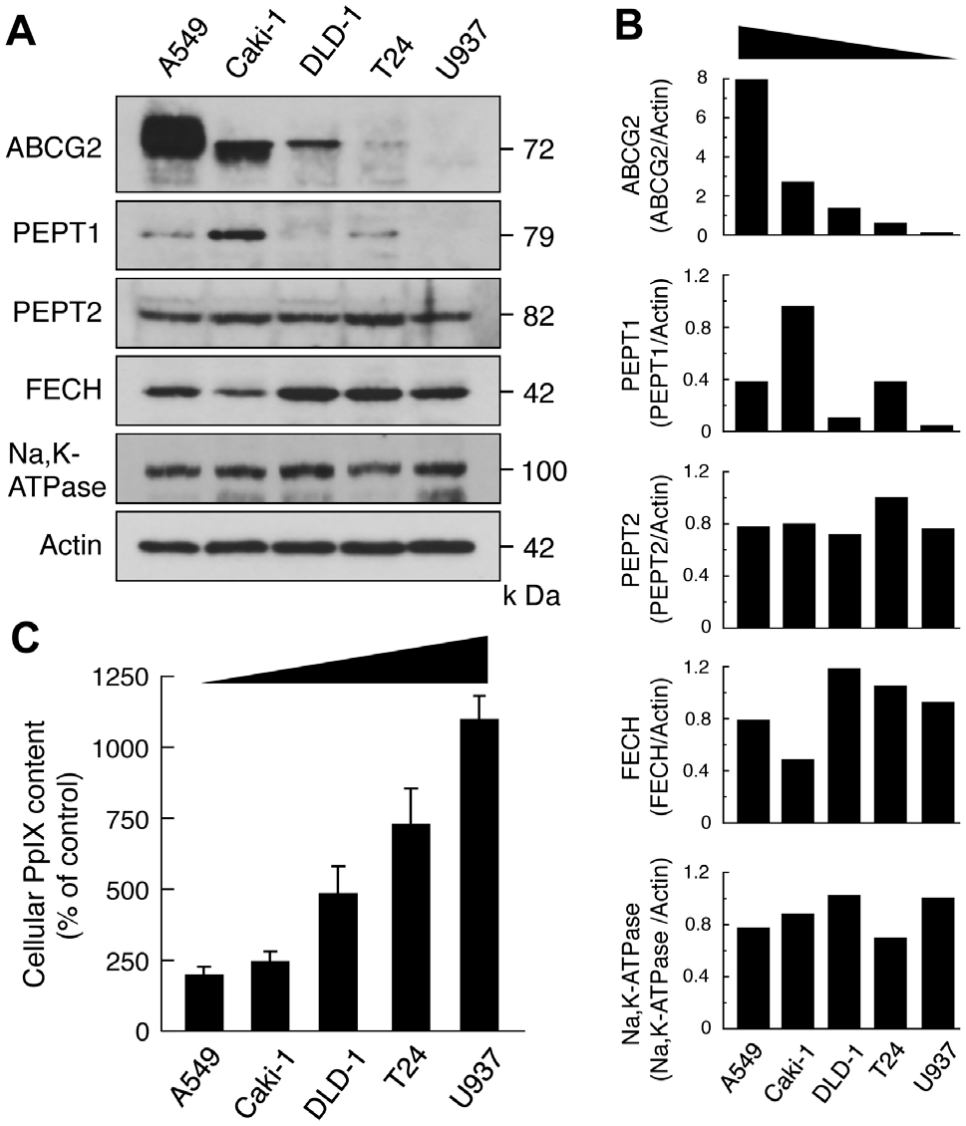
Expression of ABCG2, PEPT and FECH, and ALA-mediated PpIX accumulation in various cancer cells. Human lung adenocarcinoma cells (A549), human renal cancer cells (Caki-1),human colorectal cancer cells (DLD-1),human bladder cancer cells (T24) and human histiocytic lymphoma cells (U937) were used in this experiment. *A,* Western blotting analysis of whole cell lysates of various cancer cell lines. ABCG2 was detected with a polyclonal anti-ABCG2 (Cat. #4477; Cell Signaling Technology). Actin was used as a protein loading control. A representative blot of multiple experiments is shown. *B*, Relative contents of each protein were determined by densitometric scanning and were normalized using actin. *C*, Levels of accumulated ALA-mediated PpIX in various cancer cell lines. Bar graphs show flow cytometric analysis results of cellular PpIX content. Mean intensities of fluorescence (autofluorescence values) obtained from control experiments of each cell line were normalized to 100%. Results represent the mean ± SD of three independent experiments.

## Materials and Methods

### Reagents

ALA was purchased from COSMO OIL (Tokyo, Japan). Fetal bovine serum (FBS), Dulbecco’s modified Eagle’s medium (DMEM), Ko143, PpIX, proteinase K, monoclonal anti-FLAG IgG (clone M2), doxorubicin hydrochloride, poly-D-lysine hydrobromide, bovine serum albumin, and G418 were obtained from Sigma-Aldrich (St. Louis, MO, USA). FuGENE HD transfection reagent and endoglycosidase H (Endo H) were from Roche Diagnostics (Mannheim, Germany). Peptide:*N*-glycosidase F (PNGase F) was from Takara Bio (Otsu, Japan). Polyclonal antibodies against PEPT1 and PEPT2 were from Santa Cruz Biotechnology (Santa Cruz, CA, USA). Polyclonal anti-SEC23-related protein A (SEC23A) antibody was from Gene Tex (Irvine, CA, USA). Polyclonal antibodies against ABCG2 (Cat. #4477), cytochrome c oxidase subunit IV (CoxIV), Na, K-ATPase, GM130, and Alexa Fluor 488-conjugated goat anti-mouse IgG were from Cell Signaling Technology (Danvers, MA, USA). Monoclonal anti-ABCG2 (clone 5D3) was from R&D Systems (Minneapolis, MA, USA). Monoclonal anti-actin antibody (clone C4) was from Millipore (Temecula, CA, USA). Polyclonal antibody against ferrochelatase was kindly provided by Dr. S. Taketani (Kyoto Institute of Technology, Kyoto, Japan) [11]. Mito Tracker Red CMXRos was from Invitrogen (Carlsbad, CA, USA). BCA protein assay kit was from Thermo Scientific (Waltham, MA, USA). All other chemicals were of analytical grade and obtained from Nacalai Tesque (Kyoto, Japan). Mito Tracker Red CMXRos and Ko143 were dissolved in dimethyl sulfoxide and stored in aliquots at −20°C until use.

**Figure 2:**
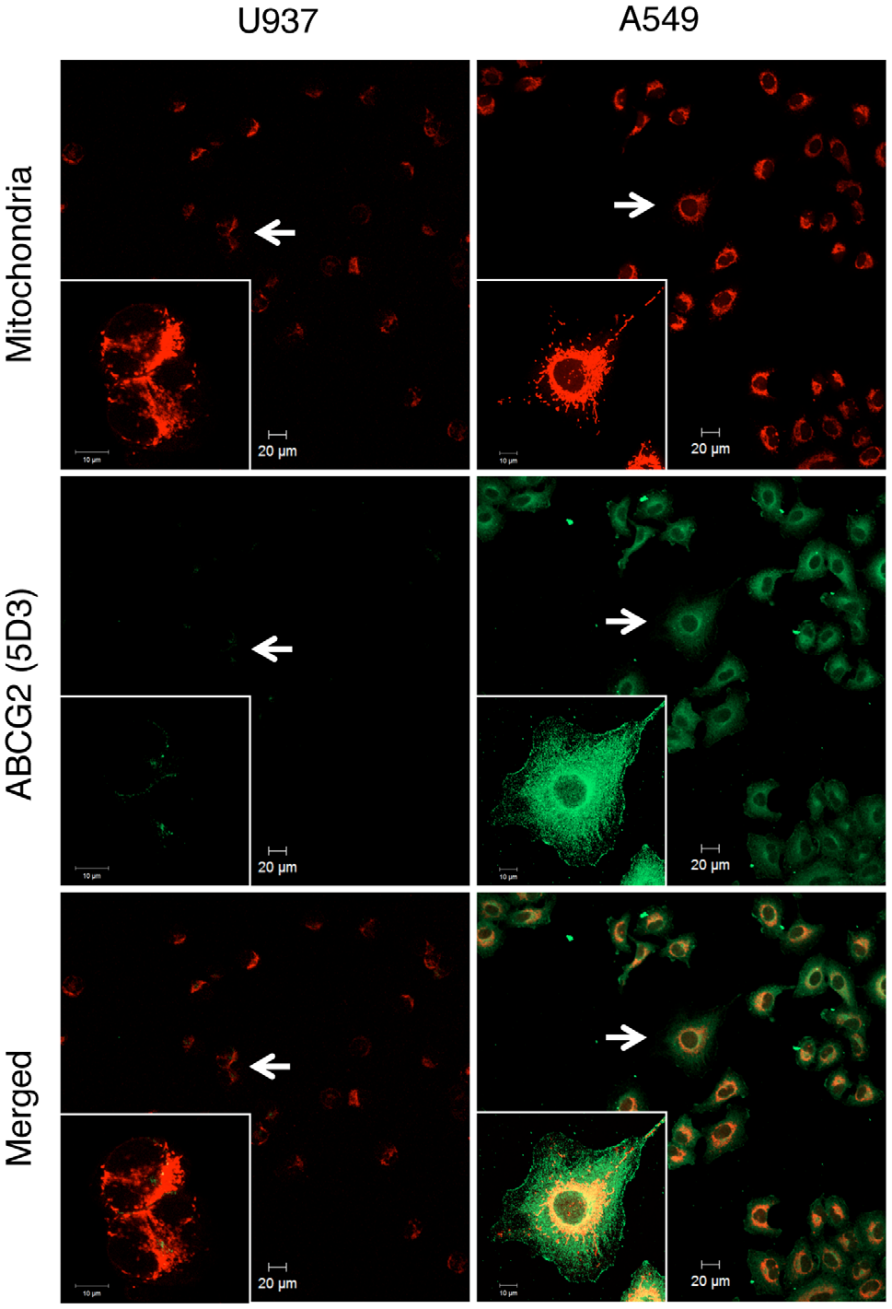
Confocal immunofluorescence microscopic analysis of ABCG2 localization. Live cells cultured on glass coverslips were incubated with 25 nM of Mito Tracker Red CMXRos to label mitochondria (*red*); then they were fixed and processed for ABCG2 protein immunostaining (*green*). Confocal images were obtained from cells immunostained with mouse monoclonal anti-ABCG2 antibody (5D3; R&D Systems) and Alexa Fluor 488-conjugated goat anti-mouse IgG antibody. Insets show a magnified area, indicated by white arrows.

### Cell Culture

A549 (a human lung adenocarcinoma cell line), Caki-1 (a human renal cancer cell line), DLD-1 (a human colorectal cancer cell line), U937 (a human histiocytic lymphoma cell line), and HEK (a human embryonic kidney cell line) cells were obtained from Health Science Research Resources Bank (Osaka, Japan). T24 (a human bladder cancer cell line) was obtained from American Type Culture Collection (Rockville, MD). Cells were maintained in complete medium; DMEM supplemented with 10% heat-inactivated FBS, and antibiotics (Invitrogen), in a humidified atmosphere of 5% CO_2_/air at 37°C. Typically, cells were seeded on a multi-well plate and cultured in the conditioned medium 24 h before each experiment [21].

**Figure 3:**
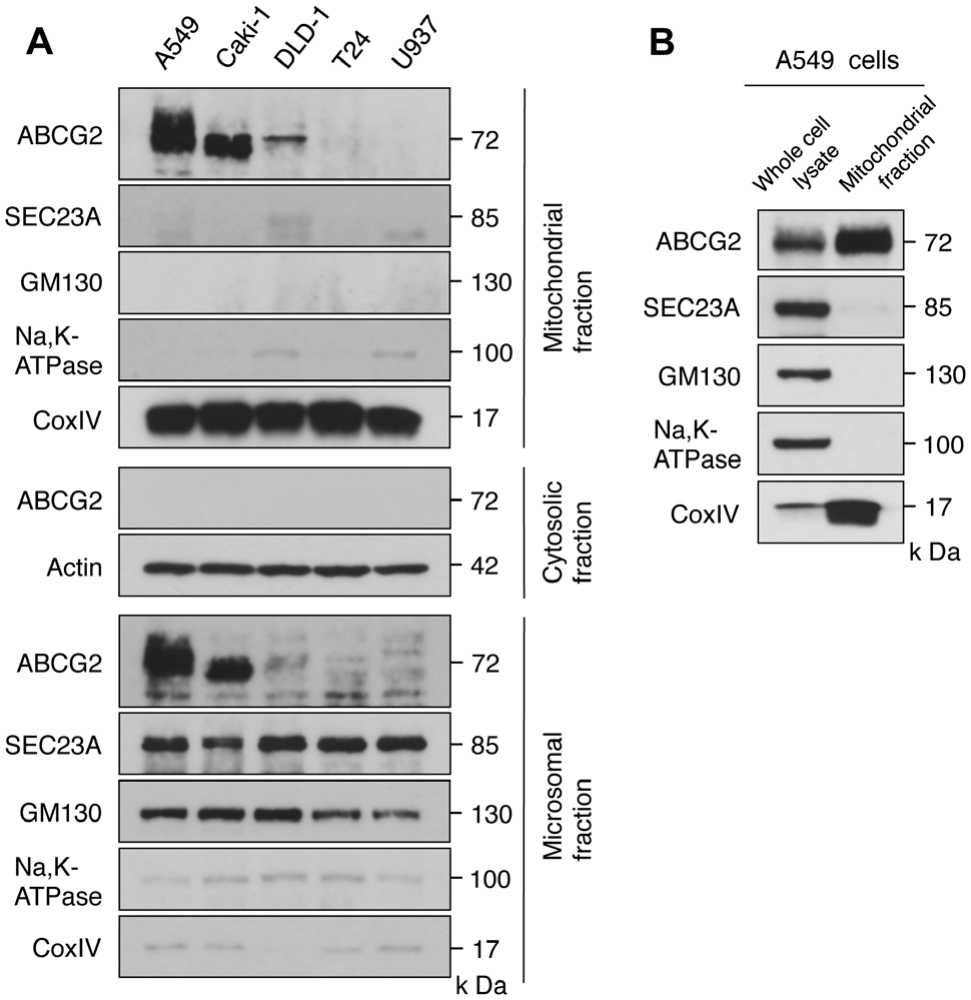
Distribution of ABCG2 in the mitochondrial fraction. *A*, Subcellular fractions were isolated by a centrifugation method as described in Materials and Methods. ABCG2 was detected by western blotting analysis using polyclonal anti-ABCG2 (Cat. #4477; Cell Signaling Technology). CoxIV, Actin, SEC23A, GM130, and Na, K-ATPase were used as organelle-specific markers of mitochondria, cytosol, endoplasmic reticulum, Golgi apparatus, and plasma membrane, respectively. A representative blot of multiple experiments is shown. *B*, Highly purified mitochondria were isolated by OptiPrep density gradient centrifugation as described in Materials and Methods and the western blotting analysis was performed using antibodies as described in A. Equal volume of samples from both whole cell lysate and mitochondrial fraction of A549 cells were subjected to SDS-PAGE. A representative blot of multiple experiments is shown.

### Plasmid Construction for N-terminal FLAG-ABCG2

Plasmid pcDNA3 FLAG-ABCG2 in which oligonucleotide coding for FLAG-tag was fused to the 5′ end of the coding sequence of ABCG2 was constructed by PCR using two oligonucleotides, (*Bam*H1-FLAG-ABCG2, 5′-ATATGGATCCATGGACTACAAGGATGACGATGACAAGTCTTCCAGTAATGTC-3′ and ABCG2-*Eco*RI, 5′-GCGCGAATTCTTAAGAATATTTTTTAAG-3′) and pF1KB5062 (Promega, Madison, WI, USA) as a template. After digestion with *Bam*H1 and *Eco*RI, the amplified fragments were subcloned into plasmid pcDNA3 at the *Bam*H1 and *Eco*RI sites [22].

**Figure 4:**
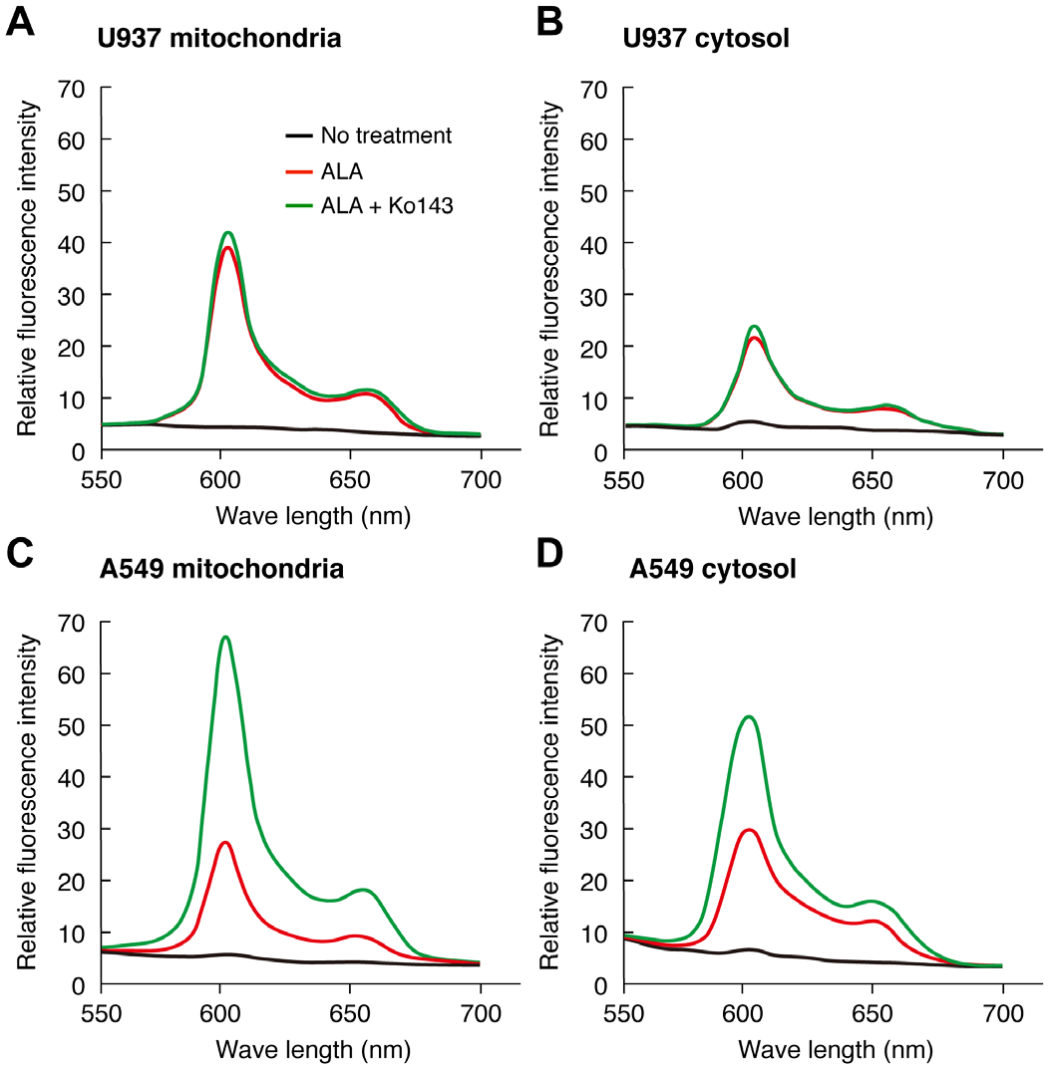
Regulation of the intra-mitochondrial content of ALA-mediated PpIX by Ko143, a specific inhibitor of ABCG2. U937 cells and A549 cells were incubated for 3 h with 1 mM ALA in complete medium in the presence or absence of 1 µM Ko143. After incubation, cells were washed with ALA-free medium and mitochondrial and cytosolic fractions were isolated as described in Materials and Methods. The fluorescence spectrum of each fraction was analyzed by a fluorophotometer. A representative histogram of three independent experiments is shown. The levels of PpIX were shown by black line (No treatment), red line (ALA without Ko143), green line (ALA with Ko143), respectively.

### Transfection and Selection of Cells Stably Express Transfected DNA

HEK cells were seeded in 6-well plates at a density of 1 × 10^5^ cells/well and incubated under normal conditions for 24 h. Recombinant FLAG-ABCG2 plasmid vector (1 µg/well) was introduced into HEK cells using FuGENE HD transfection reagent in antibiotic-free medium in accordance with the manufacturer’s instructions. To achieve stable transfection, 24 h after transfection, the cells were harvested by trypsinization and seeded at 1 × 10^4^ cells/dish in 100 mm tissue culture dishes in DMEM containing 10% FBS and 700 µg/ml G418 to select *neo* gene positive clones. The cells were cultured for at least for 2 further weeks with medium exchange every 2 days. Stably transfected-HEK (ST-HEK) cells growing and dividing at this concentration of G418 were used for each experiment [23].

**Figure 5:**
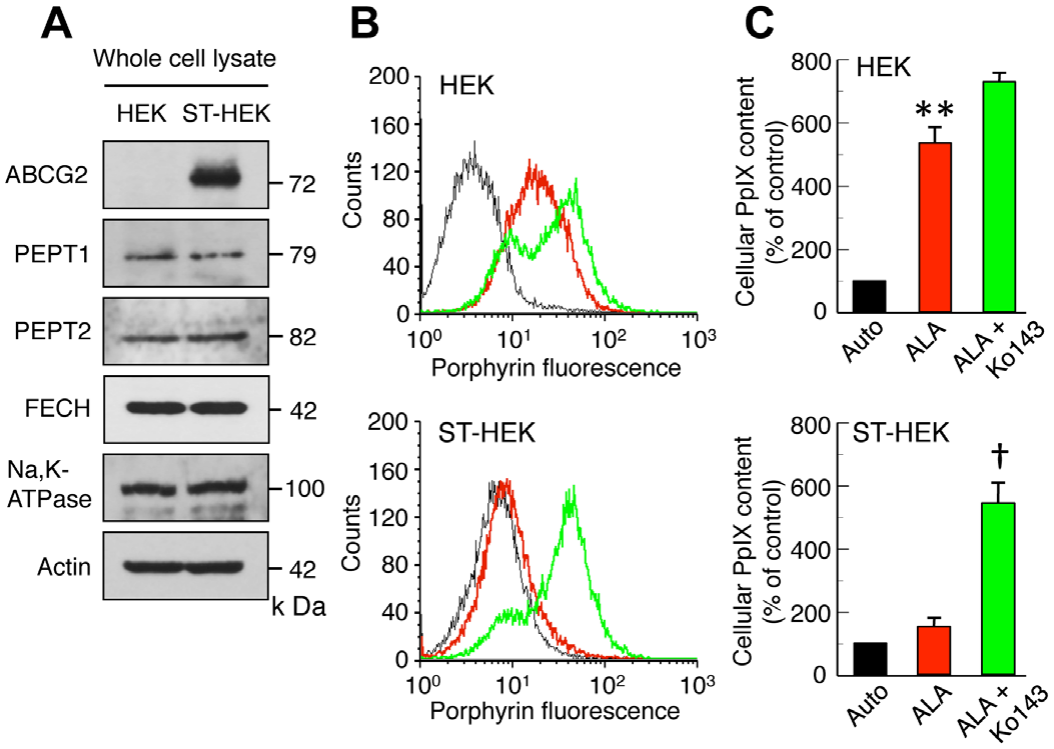
ABCG2-dependent regulation of ALA-mediated PpIX accumulation. *A*, Western blot analysis of whole cell lysates of HEK cells and ST-HEK cells. A representative blot of multiple experiments is shown. Na, K-ATPase and actin were used as a plasma membrane marker and loading control, respectively. *B*, Flow cytometric histograms of intracellular ALA-mediated PpIX contents in HEK cells and ST-HEK cells. The porphyrin autofluorescence (black line) in HEK cells and ST-HEK cells was measured. Cells were incubated with 1 mM ALA in complete medium for 3 h in the presence (*green line*) or absence (*red line*) of Ko134, and then cellular contents of PpIX were measured using a flow cytometer. *C,* Bar graphs show the flow cytometric analysis of the cellular PpIX contents. Mean intensities of fluorescence (autofluorescence values) obtained from control experiments of each cell line were normalized to 100%. Results represent the mean ± SD of three independent experiments. **P<0.01 vs. auto. †*P*<0.01 vs. ALA-treated.

### Flow Cytometric Analysis of Cellular PpIX

ALA was diluted in PBS to make a stock solution of 0.5 M and added to the cell culture medium at a final concentration of 1 mM and incubated for 3 h in DMEM containing 10% FBS. Then, the cells were washed three times with PBS and harvested by trypsinization. After centrifugation at 800 × *g* for 5 min, the cells were resuspended in 0.5 ml of PBS. Cellular PpIX contents were measured using a flow cytometer [BD Biosciences FACScan, San Diego, CA, USA (Ex. 488 nm, Em. 650 nm)] and quantified with CellQuest software (BD Biosciences) [24].

**Figure 6:**
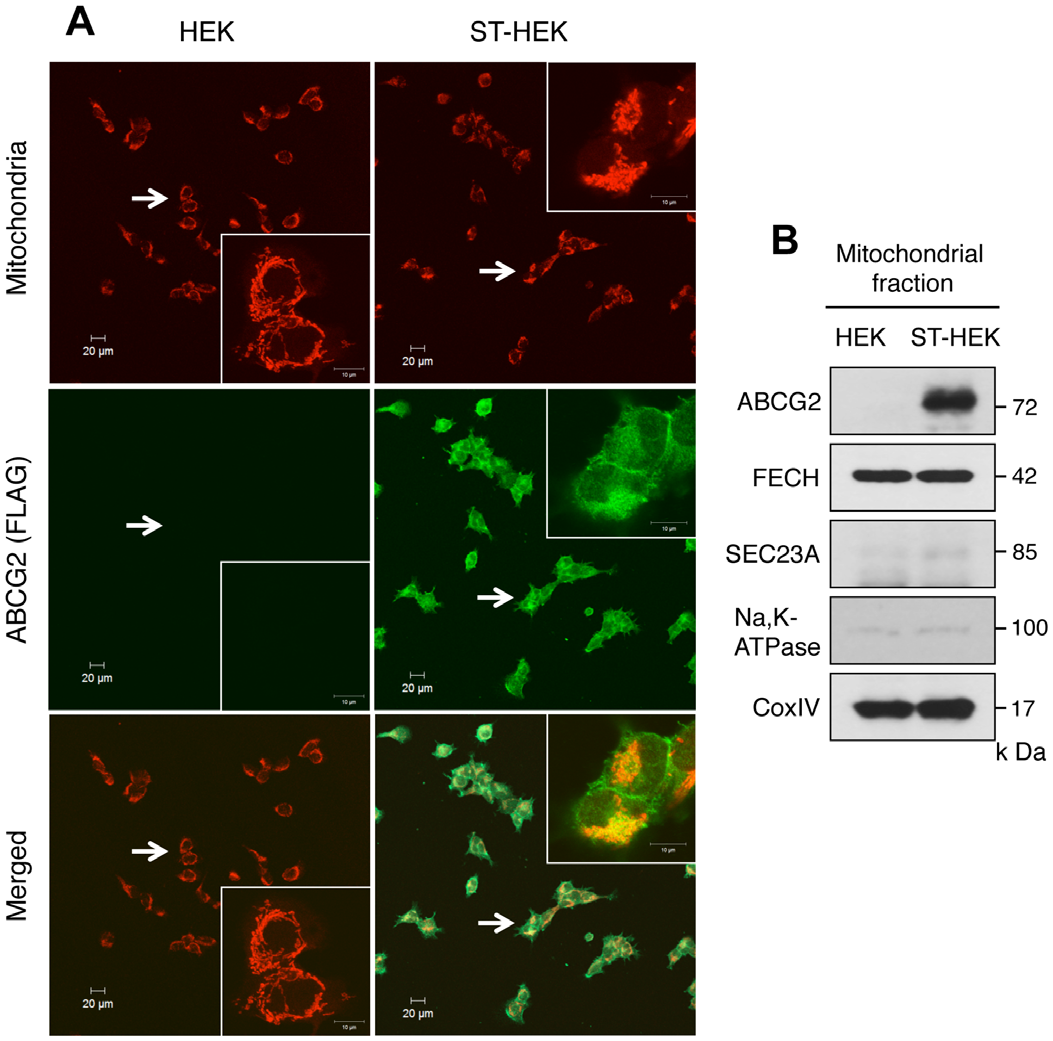
Expression and distribution of FLAG-ABCG2 in HEK cells and ST-HEK cells. *A,* Confocal immunofluorescence analysis of HEK cells and ST-HEK cells. Mitochondria, stained with Mito Tracker Red CMXRos; ABCG2, stained with monoclonal anti-FLAG IgG and Alexa Fluor 488-conjugated goat anti-mouse IgG. *B,* Western blotting analysis of mitochondrial fraction of HEK cells and ST-HEK cells. A representative blot of multiple experiments is shown.

### Confocal Immunofluorescence Microscopy

Cells grown on poly-D-lysine coated glass coverslips were incubated for 30 min at 37°C with 25 nM Mito Tracker Red CMXRos, a cell permeable mitochondria-selective dye, and fixed in 0.5% buffered paraformaldehyde for 10 min at room temperature. After washing with 10 mM glycine in PBS, the cells were permeabilized with acetone for 3 min at −20°C. Cells washed with PBS were incubated in blocking solution (0.5% bovine serum albumin and 2% glycerol in PBS) for 30 min and then with mouse monoclonal anti-ABCG2 antibody (1∶50; 5D3, R&D Systems) or with mouse monoclonal anti-FLAG antibody (1∶100; M2, Sigma-Aldrich) for 90 min at room temperature. The immunoreactions were revealed by incubation of the cells with Alexa Fluor 488-conjugated goat anti-mouse IgG. Negative control experiments were carried out by replacing the primary antibodies with non-immune goat serum. The cross-reactivity of the secondary antibodies was tested in control experiments in which the primary antibodies were omitted. Finally, coverslips containing the immuno-labeled cells were mounted with SlowFade Gold antifade reagent (Invitrogen) and observed using a Zeiss laser scanning microscope 510 equipped with a HeNe/Ar laser source for fluorescence measurements in accordance with methods described in detail previously [25]. The observations were performed using a Zeiss Plan Apochromat 20×/0.8NA or a Zeiss C-Apochromat 63×/1.2W korr water immersion objective. A series of optical sections (512 × 512 pixels each) were taken 1.7 µm (20×) or 1.0 µm (63×) in thickness. All images were analyzed using the LSM 510 software.

**Figure 7:**
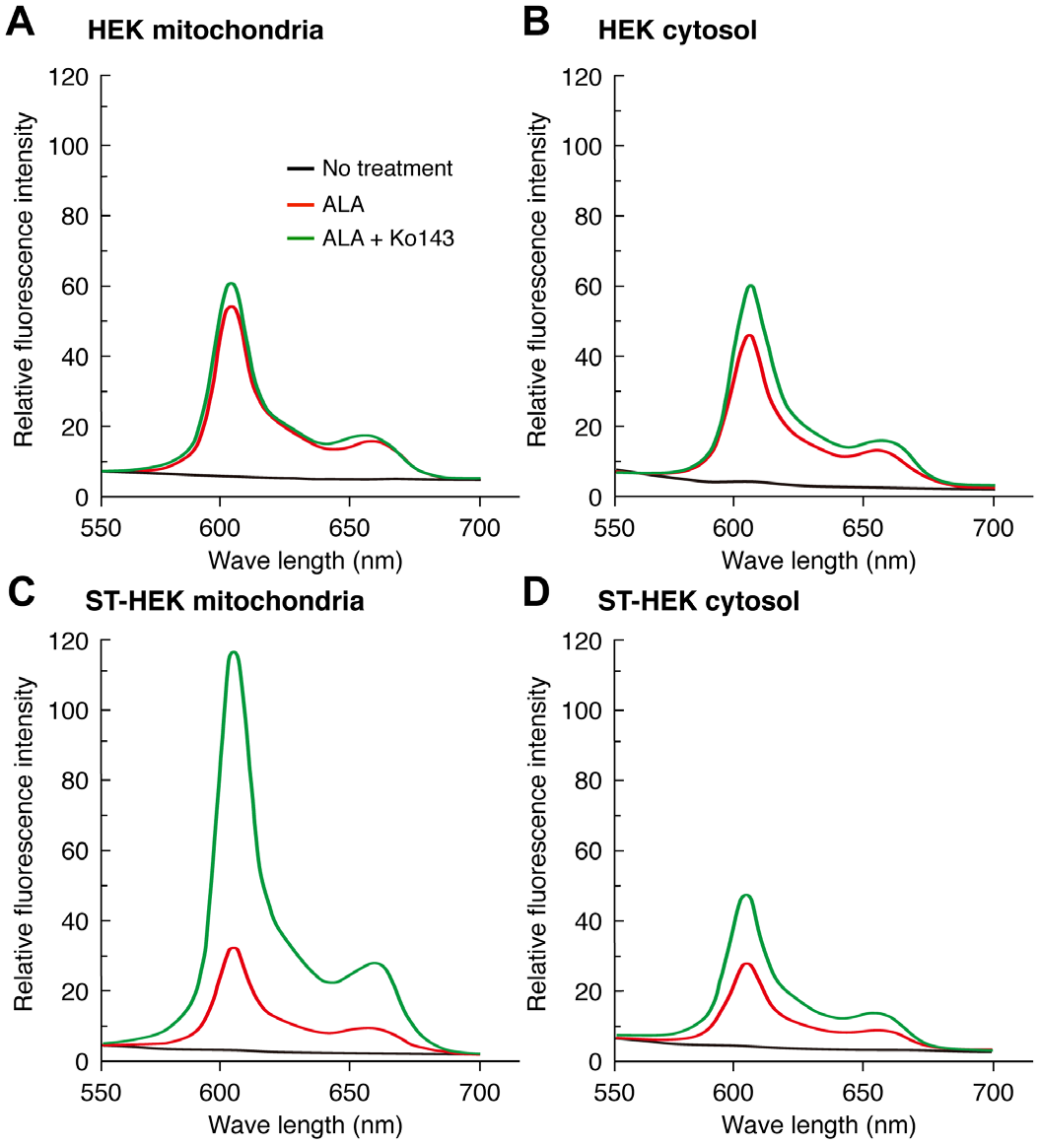
Mitochondrial ABCG2 regulates the level of ALA-mediated PpIX accumulation in mitochondria of ST-HEK cells. HEK and ST-HEK cells were incubated for 3 h with ALA in the presence or absence of 1 µM Ko143. After incubation, cells were washed with ALA-free medium and subcellular fractions were isolated as described in Materials and Methods. The fluorescence spectrum of each fraction was analyzed by a fluorophotometer. A representative histogram of three independent experiments is shown. The levels of PpIX were shown by black line (No treatment), red line (ALA without Ko143), green line (ALA with Ko143), respectively.

### Subcellular Fractionation

Subcellular fractionation was carried out by previously reported methods with modifications [26]. Briefly, cells were washed with ice-cold PBS, collected, and the pellet was suspended in 200 µl of ice-cold buffer A [20 mM HEPES (pH 7.5), 250 mM sucrose, 1.5 mM MgCl_2_, 10 mM KCl, 1 mM EDTA, 1 mM EGTA, 1 mM dithiothreitol, 1 mM phenylmethylsulfonyl fluoride, and 1 µg/ml each leupeptin, aprotinin, and pepstatin A], and homogenized in a microhomogenizer. Unbroken cells and nuclei were removed by centrifuging the homogenate at 800 × *g* at 4°C for 5 min. The resulting supernatant was subjected to 15,000 × *g* centrifugation at 4°C for 20 min. The pellet fraction (i.e., mitochondria) was washed once and suspended in ice-cold buffer A. The supernatant was recentrifuged at 100,000 × *g* for 1 h at 4°C to separate microsome and cytosol fractions, which were used in the detection of ABCG2. CoxIV and SEC23A were used as marker proteins of mitochondrial and microsomal fractions, respectively [27], [28].

**Figure 8:**
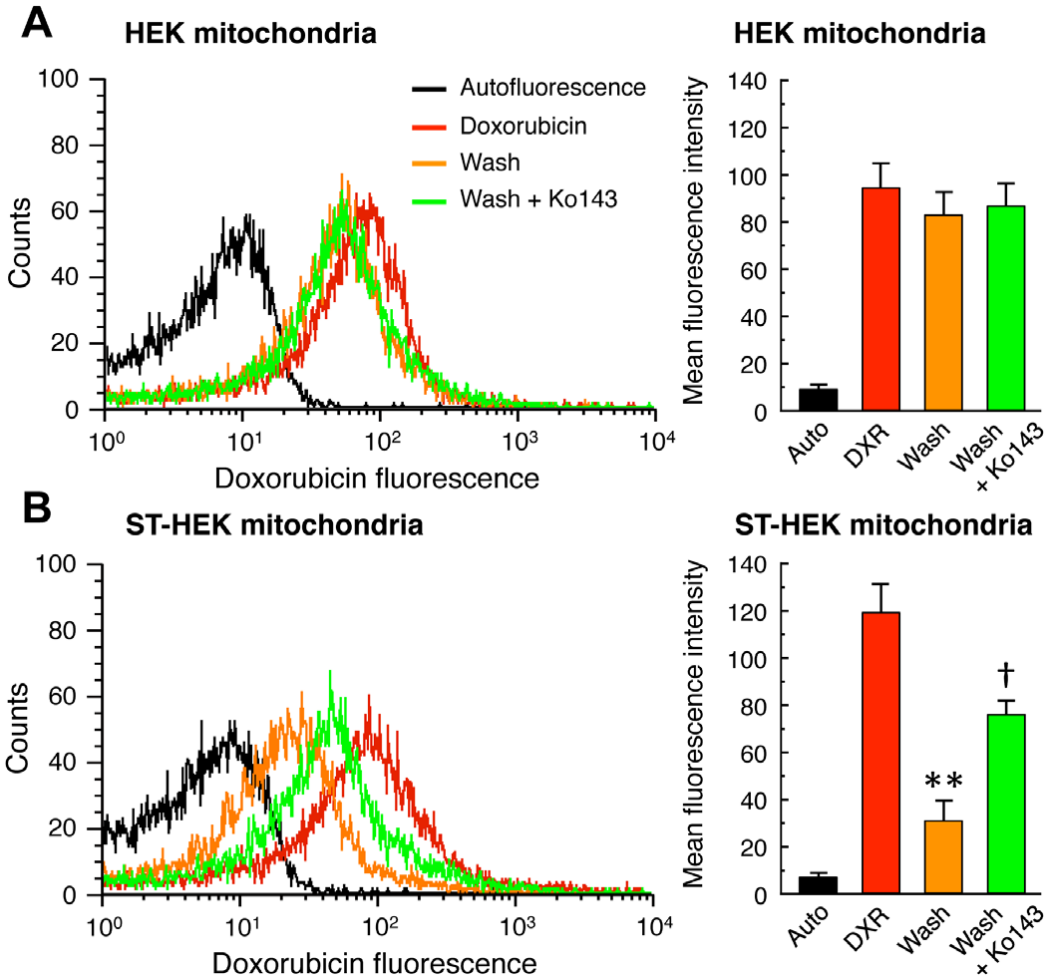
Accumulation and efflux of doxorubicin in mitochondria isolated from HEK cells and ST-HEK cells. Mitochondrial fractions were isolated as described in Materials and Methods. Mitochondrial preparations of HEK cells (A) and ST-HEK cells (B) were incubated with 5 µM doxorubicin for 10 min, then mitochondria were washed with doxorubicin-free buffer A with or without 1 µM Ko143 and incubated for 1 h. *Left panel*, contents of doxorubicin in mitochondria were analyzed using a flow cytometer. A representative histogram of three independent experiments is shown. *Right panel*, bar graphs show the flow cytometric analysis of the mitochondrial doxorubicin contents. Results represent the mean ± SD of three independent experiments. Black bar indicates autofluorescence (Auto); red bar, doxorubicin treatment (DXR); yellow bar, wash without Ko143 (Wash); green bar, wash with Ko143 (Wash+Ko143). **P<0.01 vs. DXR. †*P*<0.01 vs. Wash.

### Purification of Mitochondria by OptiPrep Density Gradient Centrifugation

Purified mitochondria were prepared by previously reported methods [29]. Cells were washed with PBS three times, resuspended in homogenization buffer [HB; 150 mM MgCl_2_, 10 mM KCl, 20 mM HEPES (pH 7.4), protease inhibitors (1 mM phenylmethylsulfonyl fluoride, 1 µg/ml each leupeptin, aprotinin, and pepstatin A); 1 ml/1 × 10^7^ cells], incubated on ice (15 min), and Dounce homogenized (35 strokes). HB with sucrose (34.2%, 1/3 vol.) was added and centrifuged at 1,000 × *g* for 5 min at 4°C to remove nuclei and unbroken cells. The supernatant was subjected to 5,000 × *g* centrifugation for 10 min at 4°C, the pellet (crude mitochondria) was resuspended in HB with sucrose (20 ml), and centrifuged at 5,000 × *g* for 10 min at 4°C. The resulting pellet was resuspended in solution A [3 ml; 20 mM HEPES (pH 7.4), 1 mM EDTA, and 250 mM sucrose]. Iodixanol solution [7.7 ml; 50% iodixanol (Axis-Shield, Oslo, Norway), 20 mM HEPES (pH 7.4), 1 mM EDTA] was added (final concentration of 36%), placed in a centrifuge tube, overlaid with solution B [10 ml; 30% iodixanol, 20 mM HEPES (pH 7.4), 1 mM EDTA], then solution C [to top, 10% iodixanol, 20 mM HEPES (pH 7.4), 1 mM EDTA], and centrifuged at 50,000 × *g* for 4 h at 4°C using swinging bucket rotor (SW28, Beckman, Palo Alto, CA). Mitochondrial fraction was collected at the 30%/10% iodixanol interface, an equal volume of solution A was added and then centrifuged at 30,000 × *g* for 10 min at 4°C. The resulting pellet was resuspended in mitochondrial suspension buffer [250 mM sucrose, 20 mM HEPES (pH 7.4) with protease inhibitors (1 mM phenylmethylsulfonyl fluoride, 1 µg/ml each leupeptin, aprotinin, and pepstatin A)]. After protein content was determined, the purified mitochondrial solution was mixed with 2 × SDS buffer and then boiled for 5 min. The samples were stored at −80°C until use.

**Figure 9:**
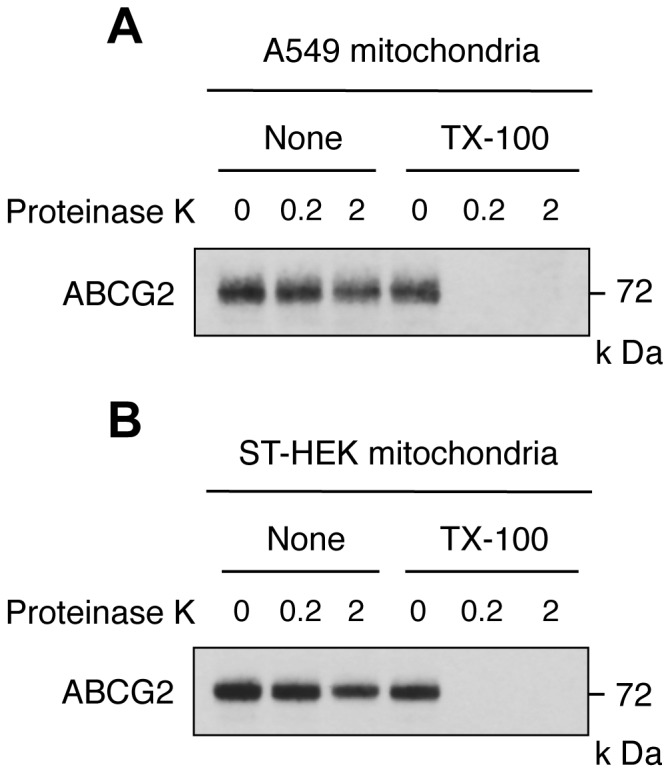
Identification of the ABCG2 distribution in mitochondrial sub-compartment. Mitochondrial preparations of A549 cells (A) and ST-HEK cells (B) were treated with proteinase K on ice for 60 min in the presence or absence of 0.2% Triton X-100 (TX-100). Then the proteins were analyzed by Western blotting using a polyclonal anti-ABCG2 antibody. A representative blot of three independent experiments is shown.

### Fluorescence Spectrophotometric Assay of PpIX Content in Mitochondrial and Cytosolic Fractions

U937, A549, HEK and ST-HEK cells were incubated for 3 h in culture medium containing 1 mM ALA in the presence or absence of 1 µM Ko143. Afterwards, the cells were washed twice with buffer A, and the mitochondrial and cytosolic fractions were obtained as described in the previous section. Then, the PpIX contents in both fractions were measured spectrofluorometrically as reported previously but with modifications [30]. Briefly, 100 µl of cytosolic fraction (300 µg of protein) was mixed with 900 µl of methanol-perchloric acid (1∶1 mixture of methanol: 0.9 M perchloric acid) and precipitated protein was removed by centrifugation. The supernatant was used for fluorescence measurements. Mitochondrial fraction (300 µg of proteins/100 µl) was disrupted in 100 µl of detergent solution (0.1M NaOH containing 0.1% SDS). After 1 h of incubation, the solution was mixed with 800 µl of methanol-perchloric acid and precipitated protein was removed by centrifugation. The supernatant was used for the analysis. Fluorescence emission spectra were recorded from 550 nm to 750 nm with an excitation wavelength of 400 nm using a Hitachi 650-10S spectrofluorometer (Tokyo, Japan). The bandwidths of excitation and emission slits were 5 nm and 2 nm, respectively. The relative fluorescence intensity at 605 nm was used for analysis of PpIX after normalization using the protein content of each sample. The cell number of different cell lines was kept approximately equal in each experiment.

**Figure 10:**
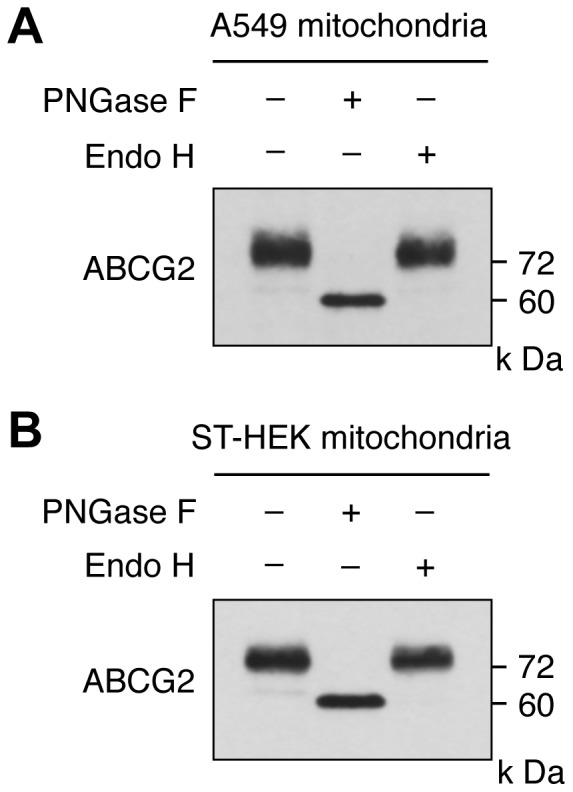
Characterization of protein *N*-glycosylation occurring on mitochondrial ABCG2 of A549 cells and ST-HEK cells. Mitochondrial preparations of A549 cells (A) and ST-HEK cells (B) were treated with PNGase F or Endo H, and then ABCG2 was detected by Western blotting using a polyclonal anti-ABCG2 antibody. A representative blot of three independent experiments is shown.

### Assay of Doxorubicin Efflux from Mitochondria by Flow Cytometry

Mitochondria isolated as described in the previous section were suspended in buffer A and kept on ice until the experiment was performed. Whole isolated mitochondria from HEK and ST-HEK cells were divided in test tubes to evaluate mitochondrial autofluorescence as well as the efflux of doxorubicin, as reported previously but with modifications [20], [31]. To estimate doxorubicin efflux, mitochondria were incubated with 5 µM of doxorubicin for 10 min at room temperature, avoiding light exposure, and then centrifuged for 5 min at 15,000 × *g* at 4°C. After washing with ice-cold buffer A, the mitochondria were suspended in l ml of buffer A and incubated for another 1 h at room temperature to allow doxorubicin efflux from mitochondria and then subjected to flow cytometry. When assessing the ABCG2-mediated doxorubicin efflux, Ko143 was added to buffer A at a concentration of 1 µM for the ABCG2-inhibited control. The doxorubicin content in mitochondria was assayed using a FACScan flow cytometer (Ex. 488 nm, Em. 575 nm) and quantified with CellQuest software. At least three independent experiments were carried out for each assay.

**Figure 11:**
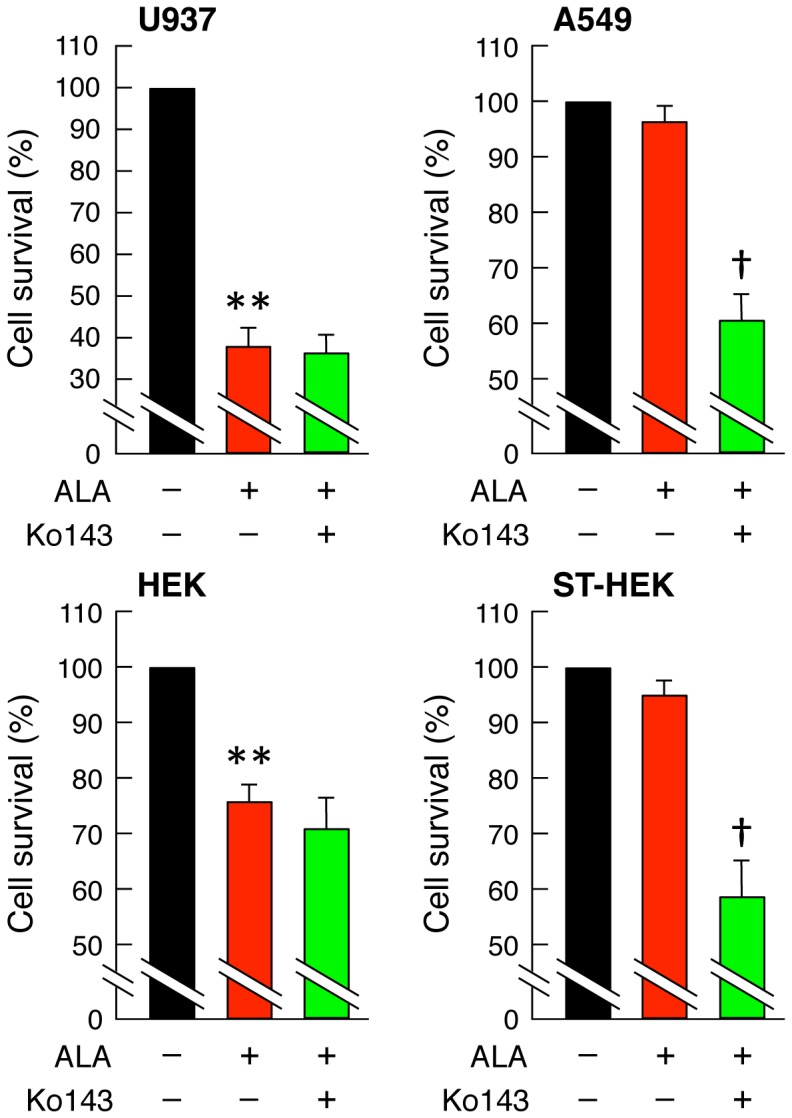
Effect of Ko143 on the cytotoxic damage of ALA-treated cancer cells by photodynamic treatment. Cells were exposed to light (29 mW/cm^2^) for 10 min using a Na-Li lamp after 3 h treatment with 1 mM ALA in the presence or absence of 1 µM Ko143. Twenty-four h after irradiation, the MTT assay was performed. Results represent the mean ± SD of three independent experiments. **P<0.01 vs. control. †*P*<0.01 vs. ALA-treated.

### Western Blotting Analysis

Cells were homogenized in ice-cold lysis buffer (20 mM Tris-HCl, pH 7.4, 0.15 M NaCl, 1% NP-40, 0.1% SDS, 0.1% sodium deoxycholate, 5 mM EDTA, 5 mM EGTA, 1 mM phenylmethylsulfonyl fluoride, and 1 µg/ml each leupeptin, aprotinin, and pepstatin A). After centrifugation of the homogenate at 15,000 × *g* for 15 min to remove the cell nuclei, the supernatant was mixed with 2 × SDS buffer and then boiled for 5 min. The samples were stored at −80°C until use. Protein content was determined using a BCA protein assay kit. The samples were subjected to SDS-polyacrylamide gel electrophoresis and proteins in the gel were transferred onto an Immobilon polyvinylidene difluoride membrane (Millipore). The membrane was blocked with 5% skim milk in TBST (0.15 M NaCl, 0.05% Tween 20, 10 mM Tris-HCl, pH 7.4), and then incubated overnight with primary antibodies diluted in 5% skim milk-TBST at 4°C. After washing three times with TBST, the membrane was incubated for 1.5 h with an HRP-conjugated secondary antibody diluted in 5% skim milk-TBST at room temperature. Immunoreactive proteins were visualized on Amersham Hyperfilm ECL (GE Healthcare) after treatment of membrane with Immobilon Western Chemiluminescent HRP Substrate (Millipore) [32].

**Figure 12:**
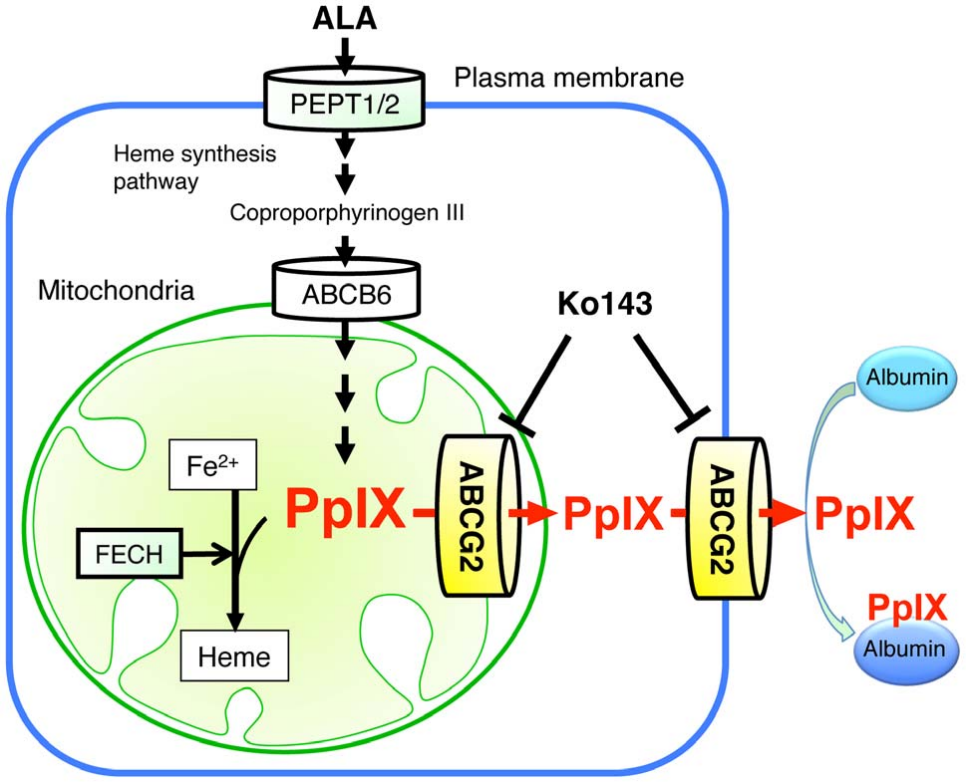
Involvement of mitochondrial ABCG2 in the regulatory mechanism of ALA-mediated PpIX accumulation in malignant cells. PEPT1/2, oligopeptide transporters; ABCB6, ABC transporter B6.

### Protease Treatment of Mitochondria

Mitochondria isolated as described in the previous section were centrifuged at 15,000 × *g* for 15 min and resuspended in buffer A without EDTA or protease inhibitors. Protease treatment of mitochondria was carried out as reported previously with modifications [33]. Briefly, limited protease digestion of mitochondria (100 µg protein/sample, protein concentration 1 mg/ml) was performed in the presence of proteinase K at a final concentration of 0.2 or 2 µg/ml (protein-to-proteinase K ratio of 5,000∶1 or 500∶1) in the absence or presence of 0.2% Triton X-100. The digestion was carried out on ice for 1 h and stopped by the addition of 4 mM phenylmethylsulfonyl fluoride. The treated mitochondria were mixed with SDS buffer and then boiled for 5 min. The samples were stored at −80°C until use.

### Glycosidase Digestion

Glycosylated ABCG2 was digested by glycosidase as described by Fukuda et al. [34]. Briefly, mitochondria isolated as described above from A549 or ST-HEK cells were denatured in 1 × denaturing buffer (0.5% SDS and 1% 2-mercaptoethanol) and incubated at 37°C for 1 h in reaction buffer (50 mM Na_2_PO_4_ (pH 7.5) and 1% Nonidet P-40) with or without PNGase F in accordance with the manufacturer’s recommendations. For Endo H digestion, the denatured mitochondria samples were incubated with or without the enzyme in 50 mM sodium citrate buffer. The reactions were stopped by the addition of 2 × SDS buffer, and the samples were subsequently subjected to SDS-PAGE and Western blotting analysis using a polyclonal anti-ABCG2 antibody.

### Photodynamic Treatment

Cells were seeded into 35 mm dish at a density of approximately 1 × 10^5^ cells per dish. Following incubation for 24 h, the cells were incubated for 3 h with 1 mM ALA in complete medium in the presence or absence of 1 µM Ko143. The cells were then exposed to light (29 mW/cm^2^) for 10 min using a Na-Li lamp (TheraBeam VR630, USHIO Inc.) [4]. The wavelength of light was 600–700 nm, preferentially 630–670 nm. Then, the cells were further cultured for 24 h. Cell survival was determined using MTT assay. Briefly, cells were incubated for 0.5 h with culture medium containing 0.5 mg/ml 3-(4,5-dimethylthiazolyl-2-yl)-2,5-diphenyl tetrazolium bromide (MTT) at 37°C. The insoluble end product (formazan derivatives) was solubilized in DMSO after removing the medium. The absorbance at 540 nm was measured using a microplate reader (Bio Rad). The cell survival was expressed as a percentage of control.

### Statistical Analysis

Statistical analysis was carried out using two-tailed *t* test (Microsoft Excel; Microsoft). The means of two distributions were considered significantly different if p<0.05.

## Results

### Relation between ALA-mediated PpIX Accumulation and Contents of ABCG2, PEPT1, PEPT2 and Ferrochelatase in Various Tumor Cell Lines

Because the expression levels of ABCG2, PEPT1, PEPT2 and ferrochelatase (FECH) are important factors that affect ALA-mediated PpIX accumulation, expression of these proteins were measured in several tumor cell lines by Western blotting analysis. Na, K-ATPase was selected as a plasma membrane marker (Fig. 1A). Protein band densities were measured and normalized using actin (Fig. 1B). Among the cells tested, A549 cells had a highest content of ABCG2, whereas U937 cells had the lowest. Notably, ABCG2 content correlated negatively with ALA-mediated PpIX accumulation, whereas the content of PEPT1, PEPT2 and FECH did not correlate with PpIX accumulation (Fig. 1A, 1B and 1C). The content of PEPT2 and FECH was not significantly different among the cell lines (Fig.1A and 1B).

### Confocal Immunofluorescence Microscopic Analysis of ABCG2 Localization in A549 and U937 Cells

After PpIX is synthesized in mitochondria, how PpIX moves from the mitochondria to the cytosol is not clearly understood. In the study of PpIX accumulation, we found that Ko143 and other inhibitors of ABC transporter affected the PpIX distribution in the cells [18]. Furthermore, it was reported recently that ABCG2 was distributed in the mitochondrial fraction of multidrug resistant (MDR) cancer cell lines [20]. Therefore, the intracellular distribution of ABCG2 in A549 and U937 cells was compared using confocal laser microscopy. The fluorescence intensity detected using an anti-ABCG2 antibody (5D3) was much higher in A549 cells than that of U937 cells, and ABCG2 was distributed in the cell membrane and in the cytoplasm, especially in the perinuclear region. Mitochondria are also localized to the perinuclear region. Confocal fluorescence microscopic analysis revealed a substantial overlap in the distribution of ABCG2 and mitochondria (Fig. 2). The merged image showed a strong yellowish-red color in A549 cells but not in U937 cells. This suggested the possibility that ABCG2 localized not only to the cell membrane but also to the mitochondria.

### Distribution of ABCG2 in the Mitochondrial Fraction

Because it was speculated that ABCG2 was distributed in mitochondria from the confocal microscopic analysis, we analyzed the sub-cellular distribution of ABCG2 in various cells by Western blotting. Using a subcellular fractionation method, we confirmed that ABCG2 was not detected in the cytosolic fraction in all cells tested. In A549 cells, ABCG2 was distributed not only in the mitochondrial fraction but also in the microsomal fraction (Fig. 3A). However, ABCG2 was not detected in the mitochondria of U937 cells. A microsomal marker protein SEC23A and a Golgi apparatus marker protein GM130 were hardly detected in the mitochondrial fraction, and the microsomal fraction has only a low level of the mitochondrial marker protein CoxIV. When highly purified mitochondria was isolated from A549 cells by OptiPrep density gradient centrifugation [29] and the western blotting analysis was performed, significant amount of ABCG2 was detected in the highly purified mitochondria, confirming the mitochondrial localization of ABCG2 as shown in Figure 3B. These results were consistent with the profile of ABCG2 distribution detected by confocal fluorescence microscopic analysis.

### ALA-mediated PpIX Accumulation in Mitochondria and its Sensitivity to Ko143

When subcellular fractionation was performed using cells pretreated with ALA, most of the PpIX was recovered in the cytosolic and mitochondrial fractions, and only a low level of PpIX was detected in the microsomal fraction irrespective of the cells used (data not shown). Therefore, we did not use the microsomal fraction to investigate the intracellular transport of PpIX. To study whether ABCG2 is involved in the efflux of synthesized PpIX from mitochondria to cytoplasm, the effect of Ko143, a selective inhibitor of ABCG2, on the level of PpIX was examined in the mitochondria and cytoplasm of cells, which were incubated in ALA-containing medium in advance. It was found that both the mitochondrial and cytosolic fractions of U937 cells contained PpIX after incubation with 1 mM ALA. The level of PpIX was higher in mitochondria than in the cytosol. Consistent with the fact that U937 has only low amounts of ABCG2, Ko143 had no effect on the level of accumulated PpIX (Fig. 4A and 4B). In contrast, in A549 cells that contain a high level of ABCG2, the levels of accumulated PpIX were not different between the mitochondrial and cytosolic fractions. Interestingly, Ko143 increased the level of PpIX both in the mitochondria and cytosol (Fig. 4C and 4D), although the mitochondrial PpIX level was slightly higher than that in the cytosolic. These results indicated that a Ko143-sensitive transporter is responsible for PpIX export from mitochondria to the cytosol in A549 cells.

### ABCG2-dependent Regulation of ALA-mediated PpIX Accumulation in ST-HEK Cells

To clarify the ABCG2 dependent regulation of ALA-mediated PpIX accumulation, we established ST-HEK cells stably transfected with a FLAG-ABCG2 plasmid vector. The protein expression of FLAG-ABCG2 detected by Western blotting in FLAG-ABCG2-transfected ST-HEK cells was very high, whereas that in parental HEK cells was negligible. However, the expression levels of PEPT1, PEPT2 and FECH were not different between these two cell lines (Fig. 5A). When Ko143 sensitive efflux of accumulated PpIX was compared between parental HEK cells and ST-HEK cells, the level of PpIX fluorescence was increased by the incubation of HEK cells with 1 mM ALA and the level was further increased by Ko143 (Fig. 5B and 5C). In ST-HEK cells, on the other hand, an increase in PpIX fluorescence was not observed by incubation with ALA, but a remarkable increase in PpIX fluorescence was detected by Ko143 treatment (Fig. 5B and 5C). These results further suggested that ABCG2 was strongly associated with intracellular PpIX accumulation.

### Expression and Distribution of FLAG-ABCG2 in HEK and ST-HEK Cells; Analysis by Confocal Fluorescence Microscopy and Western Blotting

FLAG-ABCG2 expression in HEK and ST-HEK cells was examined by confocal fluorescence microscopic analysis. The intracellular distribution of FLAG-ABCG2 was confirmed with the use of an anti-FLAG antibody. FLAG-ABCG2 distributed not only in the cell membrane but also in the cytoplasm, especially in the perinuclear region, where mitochondria also distributed predominantly (Fig. 6A). In contrast, FLAG-ABCG2 was hardly detected in HEK cells. Although the images of Mito Tracker Red and FLAG-ABCG2 were not identical, the merged image showed a substantial overlap of these fluorescence signals, which suggested that ABCG2 was expressed in mitochondria. To further confirm the distribution of the overexpressed FLAG-ABCG2 in mitochondria, a mitochondrial fraction was isolated from ST-HEK as well as HEK cells, and protein expression levels were studied by Western blotting. The mitochondrial fraction had negligible microsomal and plasma membrane contamination, as detected by a microsomal marker protein SEC23A and plasma membrane marker Na, K-ATPase, respectively. As shown in Figure 6B, FLAG-ABCG2 was detected in mitochondria from ST-HEK cells but not from HEK cells. Ferrochelatase expression was not different between the two mitochondrial preparations.

### Biological Function of FLAG-ABCG2 Distributed in the Mitochondrial Fraction of ST-HEK Cells

To study whether ABCG2 is involved in the efflux of synthesized PpIX from mitochondria to the cytoplasm, the effect of Ko143 was studied on the level of PpIX in the mitochondrial fraction isolated from HEK and ST-HEK cells. Both mitochondrial and cytosolic fractions from these cell lines showed the accumulated PpIX after incubation with 1 mM ALA for 3 h. In HEK cells, the level of PpIX in mitochondria was slightly higher than that in the cytosol. Ko143 slightly increased the level of PpIX accumulation especially in the cytosol (Fig. 7A and 7B). Levels of accumulated PpIX in the mitochondrial and cytosolic fractions in ST-HEK were lower than those in HEK cells. However, Ko143 remarkably increased the intra-mitochondrial content of PpIX, whereas no significant change was observed in the cytosolic content of PpIX (Fig. 7C and 7D). These results indicate that FLAG-ABCG2 in the mitochondrial fraction was functional in the regulation of ALA-mediated PpIX accumulation in ST-HEK cells.

### Role of FLAG-ABCG2 in Doxorubicin Transport in the Mitochondrial Fraction of ST-HEK Cells

Because ABCG2 is a well-known multidrug resistant protein, we further confirmed that the overexpressed ABCG2 was functional as a chemical transporter in the mitochondria. A mitochondrial fraction was isolated from ST-HEK cells, and incubated with doxorubicin in the presence or absence of ABCG2 inhibitor Ko143. The mitochondrial fraction accumulated doxorubicin after incubation with 5 µM doxorubicin for 10 min. These mitochondria were washed with doxorubicin-free buffer A and incubated another 1 h in the presence or absence of Ko143. After incubation with doxorubicin-free medium, accumulated doxorubicin was exported from the mitochondria from ST-HEK cells but not from those from HEK cells (Fig. 8). Moreover, Ko143 suppressed the efflux of doxorubicin from mitochondria from ST-HEK cells. These results confirmed that ABCG2 distributed in the mitochondrial fraction plays an important role in the regulation of doxorubicin accumulation in cancer cells.

### Analysis of the Localization of ABCG2 in Mitochondrial Sub-compartments

To elucidate whether ABCG2 localized on the surface of or in the intra-membrane space of mitochondria, the mitochondrial fractions from A549 cells and ST-HEK cells were treated with proteinase K in the presence or absence of Triton X-100, and ABCG2 protein was analyzed by Western blotting to determine if it was digested or not [35]. In the absence of Triton X-100, ABCG2 was resistant to proteinase K digestion. The presence of proteinase K at 0.2 ∼ 2.0 mg/ml failed to digest ABCG2. In contrast, ABCG2 was readily degraded when mitochondria were disrupted by 0.2% Triton X-100 (Fig. 9). These results indicated that mitochondrial ABCG2 is localized within the mitochondria, probably to the inner-membrane but not to the outer membrane in both A549 cells (Fig. 9A) and ST-HEK cells (Fig. 9B).

### Mitochondrial ABCG2 is Glycosylated and Traffics through the ER and Golgi

The Western blotting analysis of ABCG2 expression indicated glycosylation of ABCG2 based on the formation of a broad protein band (Fig. 1A). Therefore, we tested whether ABCG2 is modified by *N*-linked glycans. To analyze endogenous and overexpressed ABCG2, mitochondrial fractions of A549 and ST-HEK cells were treated with either Endo H, which only digests high mannose-containing oligosaccharides that remain in the endoplasmic reticulum (ER), or PNGase F, which removes high mannose as well as complex oligosaccharides from asparagine residues [34]. Immunoblotting analysis revealed lower molecular weight ABCG2 after PNGase F but not Endo H treatment (Fig. 10), indicating that ABCG2 in the mitochondrial fraction contained *N*-glycans, that have been processed in Golgi apparatus.

### Effects of Ko143 on the Cytotoxic Damage of ALA-treated Cancer Cells by Photodynamic Treatment

Effects of Ko143 on the cytotoxic damage of ALA-treated cancer cells by photodynamic treatment were studied using U937, A549, HEK, and ST-HEK cells. As a result, in U937 cells and HEK cells that contain a low level of ABCG2, cytotoxic damage of ALA-treated cells was observed by photodynamic treatment, but enhancement of cytotoxicity was not observed by Ko143 treatment as shown in Figure 11. In contrast, in A549 cells and ST-HEK cells that contain a high level of ABCG2, no obvious cytotoxic damage of ALA-treated cells was observed. However, significant enhancement of cytotoxicity was observed by Ko143 treatment (Fig. 11).

## Discussion

Accumulation of PpIX is the basis of PDD and PDT for cancer patients when ALA is supplemented exogenously. As described previously, this PpIX accumulation depends on the following three factors, i.e. ALA uptake, heme synthesis, and intra-cellular traffic of porphyrins. The definitive mechanisms of these factors were defined by detailed biochemical analysis [6]–[12]. However, the mechanism of PpIX efflux from mitochondria to the cytoplasm has not been clarified despite the fact that PpIX is synthesized inside mitochondria.

In this study, we found that ALA-mediated PpIX accumulation correlated negatively with the expression level of the PpIX transporter ABCG2, but not with that of PEPT1, PEPT2 or FECH (Fig. 1). In addition, immunofluorescence and cell fractionation studies showed that ABCG2 predominantly co-localized with mitochondria (Fig. 2). These results suggested that ABCG2 distributed in the mitochondrial fraction plays an important role in the regulatory mechanism of ALA-mediated PpIX accumulation. This interpretation was also supported by the transfection study, in which PpIX accumulation was decreased in FLAG-ABCG2 transfected HEK cells, which was reversed by the ABCG2 inhibitor, Ko143 (Fig. 5). It was also noted that the mitochondrial fraction of ST-HEK cells was sensitive to Ko143 (Fig. 7 and Fig. 8). Thus, we focused our attention on the distribution and function of ABCG2 in isolated mitochondria. As shown in Figures 2 and 6A, ABCG2 in mitochondria was not uniformly distributed, as determined by confocal immunofluorescence microscopy. We successfully separated mitochondrial and microsomal fractions without detectable cross-contamination and found that both fractions contained substantial amounts of ABCG2 (Fig. 3A, 3B).

Experimental results from proteinase K treatment of mitochondrial fractions supported the intra-mitochondrial distribution of ABCG2 (Fig. 9), because proteinase K digested ABCG2 only after the mitochondrial membrane was disrupted by Triton X-100. These results are consistent with the previous observation reported by Solazzo et al. that ABCG2 immunogold labeling of MDCK-BCRP cells (ABCG2-overexpressing MDCK cells) with anti-ABCG2 antibodies showed that both plasma membrane and mitochondrial cristae were decorated with gold particles [20].

A high level of ABCG2 expression was observed in the mitochondria isolated from A549 cells and ST-HEK cells stably transfected with FLAG-ABCG2 (Figs. 3 and 6B). When these mitochondria with a high level of ABCG2 expression were tested, ALA-induced PpIX accumulation was low. However, it increased substantially when the incubation medium contained Ko143 (Figs. 4 and 7). Furthermore, these isolated mitochondria took up exogenously added doxorubicin and released it after washing the mitochondria with doxorubicin-free incubation medium. The efflux of doxorubicin was partially inhibited by a specific ABCG2 inhibitor as reported previously [20]. These results indicated that ABCG2 localized in the mitochondria is functionally active and may contribute to the regulation of the intracellular traffic of heme and its intermediate porphyrins. From these results, we proposed a mechanism of ALA-mediated PpIX accumulation as described in Figure 12.

ABC transporters localize to the membranes of various organelles, where they play a crucial role in transport of a variety of endogenous and exogenous chemical compounds. The majority of ABC transporters localize to the ER-derived secretory organelles, including the ER, Golgi apparatus, lysosomes, and plasma membrane. These ABC transporters are initially targeted to the ER by a signal sequence, where they are integrated into the ER membrane, and then they are sorted and directed to their final destinations via vesicle transport. In the case of ABCG2, it has been demonstrated that ABCG2 localizes to the plasma membrane and exports intracellular heme and porphyrins [14].

In addition to these observations, it has been shown recently that ABCG2 is expressed in the mitochondrial cristae and that ABCG2 distributed in the mitochondria is functionally active [20]. In the present study, we also showed that ABCG2 was distributed not only in the ER-derived membranes (ER, Golgi apparatus, and plasma membrane) but also in the mitochondria of A549 cells and ST-HEK cells stably transfected with FLAG-ABCG2 (Figs. 3 and 6B). Moreover the isolated mitochondria from these cells accumulated exogenously added doxorubicin and then exported it through ABCG2 (Fig. 8). The fact that ABCG2 expressed in these isolated mitochondria is resistant to proteinase K treatment unless mitochondria are disrupted by Triton X-100 strongly suggested that ABCG2 is localized to the intra-membrane space (Fig. 9). Although ABCG2 is localized to the mitochondria, unlike other mitochondria-targeted ABC transporters such as ABCB7, ABCB8 and ABCB10 [14], [19], [36]–[39], ABCG2 lacks a canonical mitochondrial targeting sequence [40]. Interestingly, our data indicated that mitochondrial ABCG2 was glycosylated and has been processed in the Golgi apparatus. When ABCG2 in these isolated mitochondria was treated with either Endo H or PNGase F, the protein band shifted to a smaller molecular size on the Western blot only after PNGase F treatment (Fig. 10). This was the case not just in endogenous ABCG2 in A549 cells but also in stably transfected ABCG2 in ST-HEK cells. Because oligosaccharide chains become resistant to Endo H digestion after being processed in the Golgi apparatus, this result suggested that ABCG2 is cotranslationaly glycosylated and integrated into the ER-membrane and sorted to the Golgi apparatus and then finally localized in the intra-membrane space (probably inner membrane) of mitochondria. Because it is generally admitted that mitochondria are not ER-derived secretory organelles, how ABCG2 is transported from the Golgi apparatus to the intra-membrane space of mitochondria is unknown. However, a recent study revealed that the ABC transporter ABCB6, which is a mitochondrial porphyrin transporter that activates porphyrin biosynthesis, is glycosylated, suggesting that ABCB6 is synthesized on the ER membrane and then targeted to the mitochondrial membrane [34]. Therefore, an unknown mechanism that enables the transport of proteins from ER-derived organelles to the mitochondria may exist. Further analyses are required to fully elucidate the molecular mechanism of intracellular localization of ABCG2.

In this study, we investigated the role of 4 proteins, oligopeptide transporter-1 and -2, ferrochelatase, and ABCG2, in ALA-mediated PpIX accumulation and focused our attention on the function of ABCG2. However, other proteins such as porphobilinogen deaminase have been shown to affect cellular PpIX accumulation [41]. Therefore, further studies are required to understand the relative roles of ABCG2 and other enzymes/transporters in the cellular accumulation of PpIX in ALA-treated cells.
